# Modulation of mu attenuation to social stimuli in children and adults with *16p11.2* deletions and duplications

**DOI:** 10.1186/s11689-015-9118-5

**Published:** 2015-07-24

**Authors:** Caitlin M. Hudac, Anna Kresse, Benjamin Aaronson, Trent D. DesChamps, Sara Jane Webb, Raphael A. Bernier

**Affiliations:** Department of Psychiatry and Behavioral Sciences, University of Washington, 1959 Northeast Pacific Street #115, Seattle, WA 98195 USA; Seattle Children’s Research Institute, 2001 8th Avenue #400, Seattle, WA 98121 USA

**Keywords:** *16p11.2*, Copy number variation (CNV), Autism spectrum disorder (ASD), Mu attenuation, Electroencephalogram (EEG), Social perception, Molecular subtyping

## Abstract

**Background:**

Copy number variations (CNV) within the recurrent ~600 kb chromosomal locus of *16p11.2* are associated with a wide range of neurodevelopmental disorders, including autism spectrum disorder (ASD). However, little is known about the social brain phenotype of *16p11.2* CNV and how this phenotype is related to the social impairments associated with CNVs at this locus. The aim of this preliminary study was to use molecular subtyping to establish the social brain phenotype of individuals with *16p11.2* CNV and how these patterns relate to typical development and ASD.

**Methods:**

We evaluated the social brain phenotype as expressed by mu attenuation in 48 children and adults characterized as duplication carriers (*n* = 12), deletion carriers (*n* = 12), individuals with idiopathic ASD (*n* = 8), and neurotypical controls (*n* = 16). Participants watched videos containing social and nonsocial motion during electroencephalogram (EEG) acquisition.

**Results:**

Overall, only the typical group exhibited predicted patterns of mu modulation to social information (e.g., greater mu attenuation for social than nonsocial motion). Both *16p11.2* CNV groups exhibited more mu attenuation for nonsocial than social motion. The ASD group did not discriminate between conditions and demonstrated less mu attenuation compared to the typical and duplication carriers. Single-trial analysis indicated that mu attenuation decreased over time more rapidly for *16p11.2* CNV groups than the typical group. The duplication group did not diverge from typical patterns of mu attenuation until after initial exposure.

**Conclusions:**

These results indicate atypical but unique patterns of mu attenuation for deletion and duplication carriers, highlighting the need to continue characterizing the social brain phenotype associated with *16p11.2* CNVs.

**Electronic supplementary material:**

The online version of this article (doi:10.1186/s11689-015-9118-5) contains supplementary material, which is available to authorized users.

## Background

Autism spectrum disorders (ASD) are associated with a recurrent ~600 kb BP4-BP5 *16p11.2* copy number variation (CNV) [1–4]. The overall population prevalence of *16p11.2* deletions and duplications is estimated at 1/1000 [5], but their prevalence is much higher in clinical populations, with both types of CNVs accounting for approximately 1 % of ASD cases [1, 2]. Additional clinical features of *16p11.2* CNV include congenital anomalies, language delay, epilepsy/seizures, and behavioral problems such as attention deficit hyperactivity disorder [3, 5–14]. Notably, 15 % of deletion carriers meet strict diagnostic criteria for ASD [15]. Even in individuals not meeting criteria for behaviorally defined ASD diagnoses, *16p11.2* CNV confers a quantitative risk to a variety of phenotypic domains [16] and specifically to social ability [15, 17], with an observed 1.7 SD decrement in social ability relative to unaffected family members.

Given this evidence of social impairments associated with *16p11.2* CNV and the hallmark social impairments in ASD [3, 5–14, 18], the social brain is an appropriate target to elucidate the relationship between genetics and social functioning. Perception of social motion (e.g., biological motion) has been suggested as an ideal candidate neuroendophenotype [16, 19], and social motion perception may be a critical process that underlies imitation, the interpretation of goals, and social-action understanding [20, 21]. Evidence using neuroimaging suggests that individuals with ASD exhibit atypical social motion perception, including reduced activation compared to controls within the superior temporal sulcus, fusiform gyrus, and prefrontal cortex [1–4, 19, 22, 23].

Many children with neurodevelopmental disorders, including children with *16p11.2* may struggle in the fMRI scanning environment, and as such, electroencephalogram (EEG) provides a more behaviorally flexible means of assessing social neural systems. EEG mu attenuation is one such measurement of social brain function that is helpful in understanding how social difficulties relate to underlying brain function. Mu attenuation is computed as power changes in the electrophysiological mu rhythm band (8–13 Hz) in response to observed motion. Greater mu attenuation is thought to reflect desynchronization of cell assemblies in sensorimotor cortex [5, 24]. Typically developing individuals elicit mu attenuation by the execution and observation of motoric action [1, 2, 24–30]. Such findings are thought to be evidence of an execution/observation matching system [3, 5–14, 31] that contributes to many facets of social cognition including imitation, empathy, and perception of goal-directed actions [15, 32–34]. Impairments of this system have been associated with ASD and heterogeneity within the ASD diagnosis [5, 16, 35–38], suggesting that it may be sensitive to the deficits in social ability in *16p11.2* CNVs.

The aim of this study was to better characterize the functional brain phenotype of a preliminary sample of *16p11.2* CNV carriers using mu attenuation elicited by videos of social and nonsocial motion. First, group differences were evaluated between typical individuals, *16p11.2* deletion carriers, *16p11.2* duplication carriers, and individuals identified with idiopathic autism. Overall, we predicted that the *16p11.2* CNV groups would exhibit atypical mu attenuation similar to the ASD group. Second, in addition to overall group patterns, we aimed to determine how patterns of mu attenuation changed over the course of stimulus exposure for each group. Recent evidence suggests that mu attenuation may progressively change over the course of an experiment [1, 2, 15, 17, 39]. Despite known differences in information processing [3, 5–14, 18, 40–42] and neural evidence of reduced habituation in ASD to social stimuli [15, 16, 19, 43, 44], no studies have explored how mu attenuation is modulated over time in ASD. Single-trial analyses were implemented with the expectation that habituation patterns would differ between the *16p11.2* CNV groups, the ASD group, and the typical individuals. Lastly, post hoc comparisons were included to better characterize patterns of mu attenuation in the *16p11.2* CNV groups, considering the heterogeneity related to age and ASD diagnosis.

## Methods

### Participants

This study reports findings from 48 child and adult participants who were enrolled in and characterized as belonging to one of four groups: (1) deletion carrier, (2) duplication carrier, (3) idiopathic ASD, and (4) typical development. All participants spoke fluent English and had normal or corrected-to-normal vision. All research procedures conformed to regulations in accordance with the local ethical review board at the University of Washington, which approved this project. Written informed consent was obtained from each adult participant or parental representative(s). All children verbally assented to participate in the procedures, and written assent was obtained from children with a mental age of 7 or greater.

Full participant characterization is reported in Table 1. Deletion (*n* = 12) and duplication (*n* = 12) carriers were recruited following enrollment and participation in the Simons VIP Connect project [16, 20, 21, 45]. Recruitment methods included self-referral and web-based networks that directed individuals to the Simons VIP website (http://SimonsVIPconnect.org), as well as referral by clinical genetic testing centers. As part of the Simons VIP Connect, individuals were identified with the same recurrent ~600 kb 16p11.12 BP4-BP5 deletion or duplication. Within these two *16p11.2* CNV groups, no additional pathogenetic CNVs or monogenic disorders were known (see Simons VIP Consortium [45] for complete recruitment and inclusion/exclusion criteria). Individuals within the ASD and typical groups were recruited from previous projects, matched on age (ASD/typical) and verbal IQ (ASD) to the CNV groups. None of the children within the idiopathic ASD group had any likely causal CNVs, verified through array CGH sequencing [15, 17, 46].

**Table 1:** Participant characterization

Group	*N*	Age mean in years (SD)	Age range (years)	Adult:children	Gender	ASD diagnosis	FSIQ mean (SD)	VIQ mean (SD)	NVIQ mean (SD)	SRS total (SD)^a^	CNV inheritance
Deletion carriers	12	18.1 (14.0)	8–45	3:9	4 males	3 yes	88.36 (17.73)	86.00 (18.66)	91.75 (17.43)	70.36 (17.10)	4 de novo
					7 females	9 no					4 inherited
											1 germline mosaicism
											3 unknown
Duplication carriers	12	22.7 (13.8)	6–50	5:7	6 males	3 yes	87.00 (23.31)	87.92 (24.30)	87.42 (21.36)	67.17 (17.7)	1 *de novo*
					6 females	9 no					7 inherited
											4 unknown
ASD	8	11.5 (3.3)	7–15	0:8	4 males	11 yes	110.5 (18.97)	107.25 (15.17)	110.88 (20.06)	77.50 (12.84)	–
					4 females						
Typical	16	16.2 (10.5)	8–43	4:12	9 males	16 no	119.56 (7.11)	121.63 (10.15)	113.25 (8.58)	44.00 (3.79)	–
					7 females						

One deletion carrier excluded from FSIQ and VIQ characterization due to English as second language. One duplication carrier IQ scores reported as a ratio between mental and chronological age because standard scores were unavailable

*ASD* autism spectrum disorders, *SD* standard deviation, *FSIQ* full-scale IQ, *VIQ* verbal IQ, *NVIQ* nonverbal IQ, *CNV* copy number variation, *SRS* social responsiveness scale

^a^Missing data from deletion carrier (*n* = 1), typical group (*n* = 4)

For ASD and *16p11.2* CNV groups, diagnosis of ASD was confirmed using the Autism Diagnostic Interview-Revised [3, 5–14, 18, 47] and the Autism Diagnostic Observation Schedule [16, 19, 48]. IQ was assessed using the Wechsler Abbreviated Scale of Intelligence [20, 21, 49] or the Differential Ability Scales-Second edition [1–4, 19, 22, 23, 50], depending on age. One-way analysis of variance (ANOVA) indicated that there were no differences between groups in age, *F* (3,44) = 1.59, *p* = 0.21. The duplication, deletion, and the ASD groups did not differ in verbal IQ or SRS total score, *F*s (2,29) < 2.96, *p*s > 0.068, but did differ in full-scale and nonverbal IQ, *F*s (2,29) > 3.68, *p*s < 0.038. Least squares post hoc comparisons indicated that the ASD group had higher full-scale IQ and nonverbal IQ than both *16p11.2* deletion (FSIQ, SE = 22.14, *p* = 0.027; NVIQ, SE = 19.13, *p* = 0.041) and *16p11.2* duplication (FSIQ, SE = 23.50, *p* = 0.018; NVIQ, SE = 23.46, *p* = 0.014) groups. None of the typical participants had elevated scores on the SRS nor met diagnostic criteria for any psychiatric disorders.

### Social motion task

**Fig. 1:**
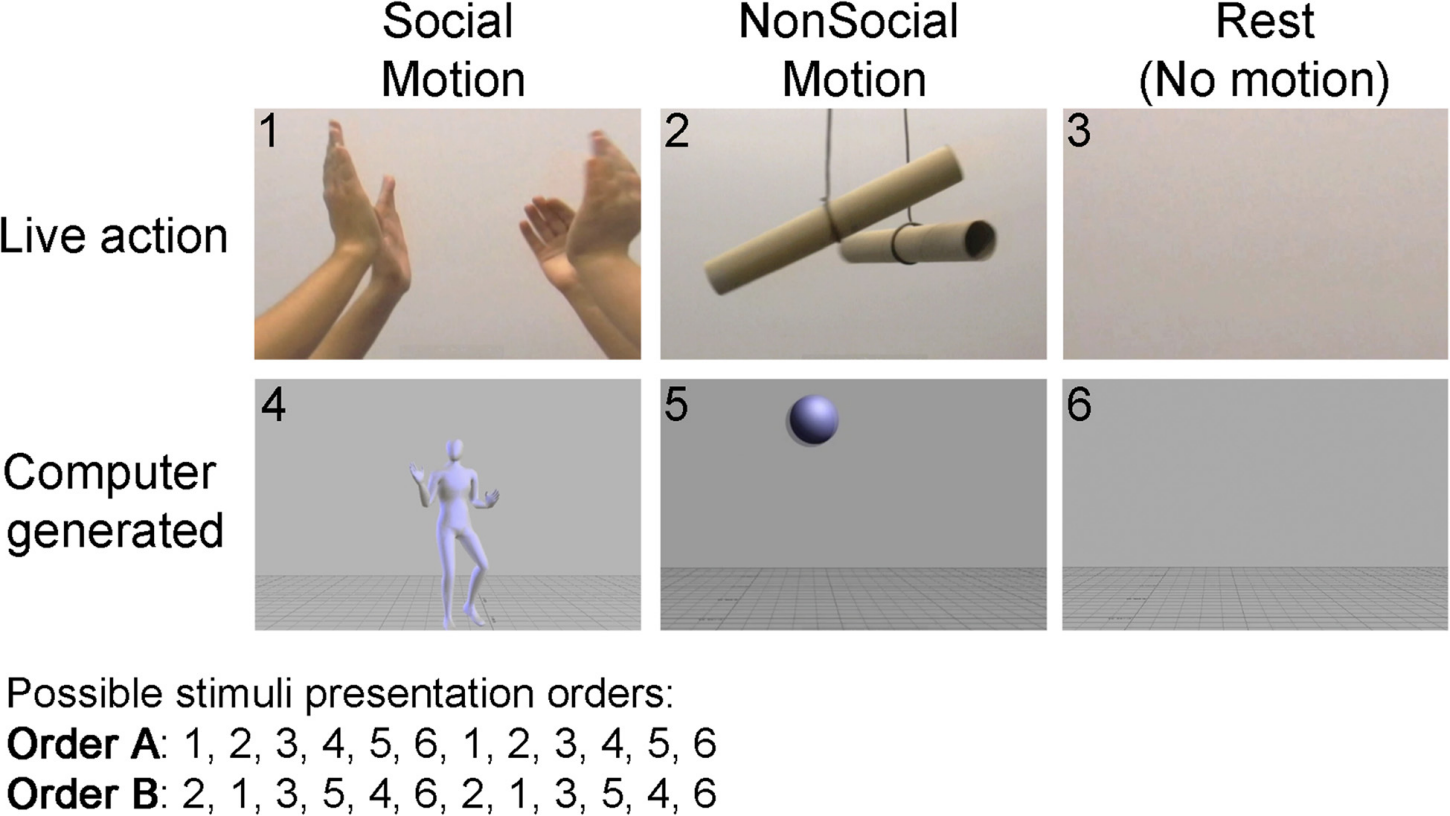
Still frames of stimuli. Participants watched live action and computer-generated videos for each condition (social, nonsocial, rest)

Participants observed a series of silent 1-min-long videos. Stimuli consisted of three conditions: (1) the social motion condition included a video of a faceless computer-generated (CG) avatar dancing and a live action (LA) video of two pairs of hands engaged in a clapping game. The computerized dancer stimuli were provided courtesy of Nick Neave and Kristofor McCarty at Northumbria University. (2) The nonsocial motion condition included a video of a CG bouncing ball and a LA video of cardboard tubes swinging. (3) The rest condition consisted of static image of the same background as the videos (Fig. 1). The quantity of visual movement was controlled across social and nonsocial motion conditions. Background complexity was controlled across all conditions. All videos were presented twice, so that each participant observed eight 1-min movement videos (four social, four nonsocial) and four 1-min rest videos. All participants saw the LA videos first followed by the CG videos, and the order of video context (i.e., social or nonsocial) was counterbalanced across participants (see Fig. 1 for possible presentation orders). Between videos, participants were directed to take a break and the experimenter initiated the next stimulus after confirmation that the participant was ready. Participants were seated approximately 75 cm from a video monitor and were instructed to sit still and attend to the videos. Video stimuli were displayed using E-Prime 2.0 software (Psychology Software Tools, Inc. Pittsburgh, PA) at a size of 27 cm by 36.8 cm and subtended a visual angle of 20.4° by 27.6°.

### Electrophysiological recording and preprocessing

**Fig. 2:**
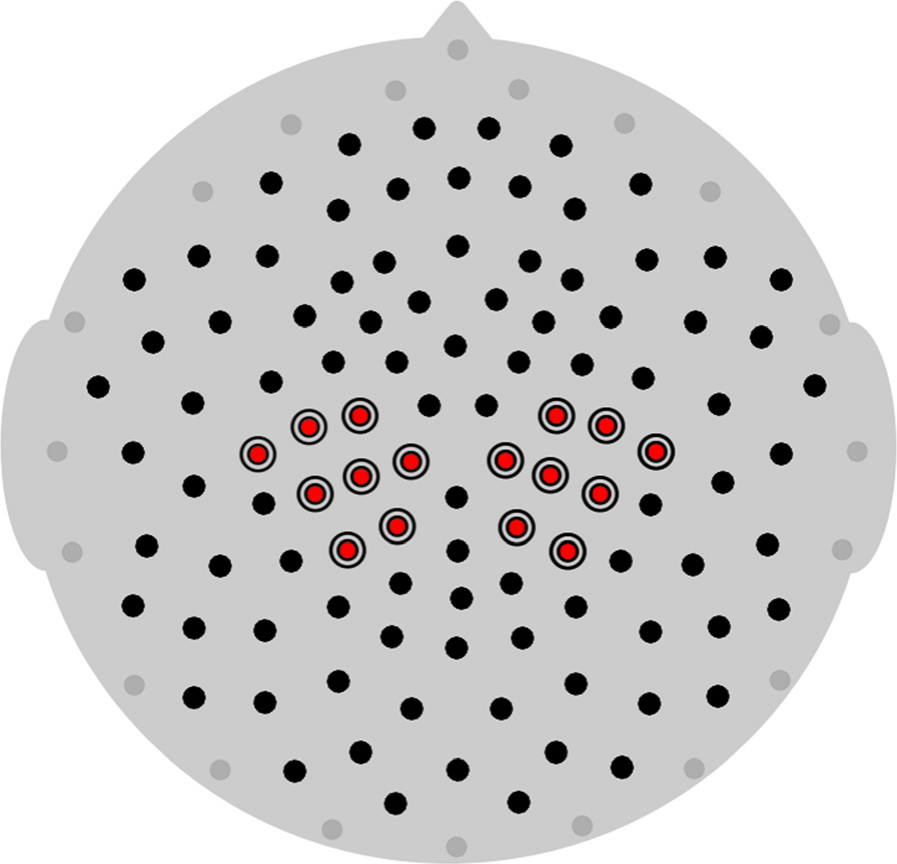
Central electrode locations. Central electrodes on the EGI Geodesic 128-channel high-density geodesic net are indicated in *red*. Rim electrodes excluded from post-processing and subsequent analysis are indicated in *gray*

Continuous EEG was recorded from a high-density 128-channel geodesic net using Net Station 4.3.1 software integrated with a 200-series high-impedance amplifier (Electric Geodesics Inc, Eugene, OR). Electrode impedances were below 50 kΩ to maximize signal-to-noise ratio, within the standard range for high-impedance amplifiers. During collection, EEG signals were referenced to the vertex electrode, analog filtered (0.1 Hz high-pass, 100 Hz elliptical low-pass), amplified, and digitized with a sampling rate of 500 Hz. A photocell recorded and marked the precise onset time of each video. During acquisition, researchers observed and marked periods containing movement and/or improper attention (e.g., participant looking away). As electrodes surrounding the face, eyes, and rim of the net are prone to artifacts, we excluded these electrodes in all participants from all subsequent post-processing or analyses (see Fig. 2 for locations).

Following data collection, data was filtered using a high-pass filter of 1 Hz. Continuous EEG was segmented into 2-s epochs starting with the onset of each 1-min video (as marked by the photocell). Epochs marked during acquisition as contaminated by movement or improper attention were removed. Automatic artifact detection rejected channels containing voltage shifts greater than 100 μV for each epoch. If the channel was rejected for more than 50 % of epochs, the channel was excluded from analysis. Epochs were re-referenced to the average reference and exported as a concatenated file per participant for additional artifact rejection using independent components analysis (ICA) via EEGLAB 13.2.1 [5, 24, 51]. First, the ICA runica algorithm was implemented across the entire concatenated dataset for each participant. Epochs related to artifacts were rejected manually. A second ICA with the runica algorithm was implemented to reject components related to artifacts. The resultant primary independent components (i.e., first 35) containing horizontal and vertical eye and/or body movements were manually inspected. Independent components containing artifacts were rejected and removed from the data. The remaining data for each condition was segmented into 2-s epochs for analysis.

To calculate mu power, fast Fourier transforms (FFTs) were conducted in Matlab (version 7.12.0, R2011a; Natick, MA) on each 2-s epoch. The power spectra occurring between 8 and 13 Hz was averaged across central electrodes clustered around the standard C3 (electrode 37) and C4 (electrode 104) positions (see Fig. 2 for locations). Power spectra for each epoch per condition were considered relative to the average rest condition power. Mu attenuation was computed as the natural log of the ratio between power for each epoch of either social or nonsocial motion minus the average power of the rest condition for that individual. Subsequently, zero represents no mu attenuation (social motion − rest = 0) and larger negative values represent more mu attenuation (i.e., social motion < rest).

### Data analysis strategies

All analyses were conducted via SAS 9.3 (SAS Institute) using restricted maximum likelihood (REML) and Satterthwaite denominator degrees of freedom. To fully assess group condition differences, multilevel models were generated using PROC MIXED to describe the variances and covariances of mu attenuation. All models included a random intercept for each individual. Of the 48 participants, there were 36 unique families enrolled in this study. To account for possible shared variance between family members, statistical analyses included random intercepts for each family. Post hoc comparisons were conducted using least squares differences. Significant effects are presented for *p* < .05; marginal effects are presented for *p* = .06 to .1.

Two different strategies were implemented. First, overall group mu attenuation differences were assessed as an average value across all epochs for each condition (model 0, model 1). Effects of condition were separated by context (0 = nonsocial, 1 = social) and environment (0 = live action, 1 = computer generated). Additional subject predictors were included in model 1 as fixed effects to test additional contribution by each individual’s age and gender, particularly as carrier and typical groups consisted of both children and adults. Age was centered at 9 years due to the fact that the majority of individuals were between 6 and 18 years of age (*n* = 36) with an average age of 8.6 years. A categorical binary variable were used for gender (0 = male, 1 = female). In addition, a predictor describing the number of epochs contributed by each individual was added as a fixed effect to determine whether the results were influenced by the overall amount of data for each person. Effects were added simultaneously initially to model 0, and nonsignificant effects were removed to generate model 1.

Second, we were also interested in whether mu attenuation varied across the course of the video. To validate the necessity for this analysis, interclass correlations calculated from an empty model indicated that 46.7 % of the variance for mu attenuation is due to overall within-person effects, while the majority of variance (53.3 %) is due to trial order. This indicates that mu attenuation is related to changes across the course of the video, such that a fixed effect of time (e.g., temporal order for each epoch) merits addition to the multilevel models. Time was centered at epoch 30 (i.e., the 30th epoch surviving epoch rejection due to artifacts). Thus, model 2 included predictors from model 1 with time added as a fully interacting predictor with context, environment, and group.

## Results

### Averaged data (model 0)

A full factorial design between context, environment, and group with age, gender, and number of epoch predictors was estimated in model 0. First, age significantly contributed to model 0, *F* (1,31.9) = 5.11, *p* = 0.031, suggesting that older individuals are predicted to exhibited incrementally greater mu attenuation (0.015 more mu attenuation for every year older than 9 years). Second, mu attenuation was not affected by gender, *F* (1,40.9) = 0.23, *p* = 0.62, indicating that female and male individuals exhibit equivalent patterns of mu attenuation within groups and across conditions. Third, the number of epochs contributed by each individual also did not contribute to patterns of mu attenuation, *F* (1,40.9) = 0.42, *p* = 0.52. This was important to determine since the ASD group contributed fewer trials due to having more trials rejected by artifacts (30.8 %) than any of the other groups (deletion carriers, 14.4 %; duplication carriers, 11.3 %; typical, 9.7 %). Thus, gender and number of epoch predictors were subsequently removed for model 1.

### Averaged data (model 1)

**Fig. 3:**
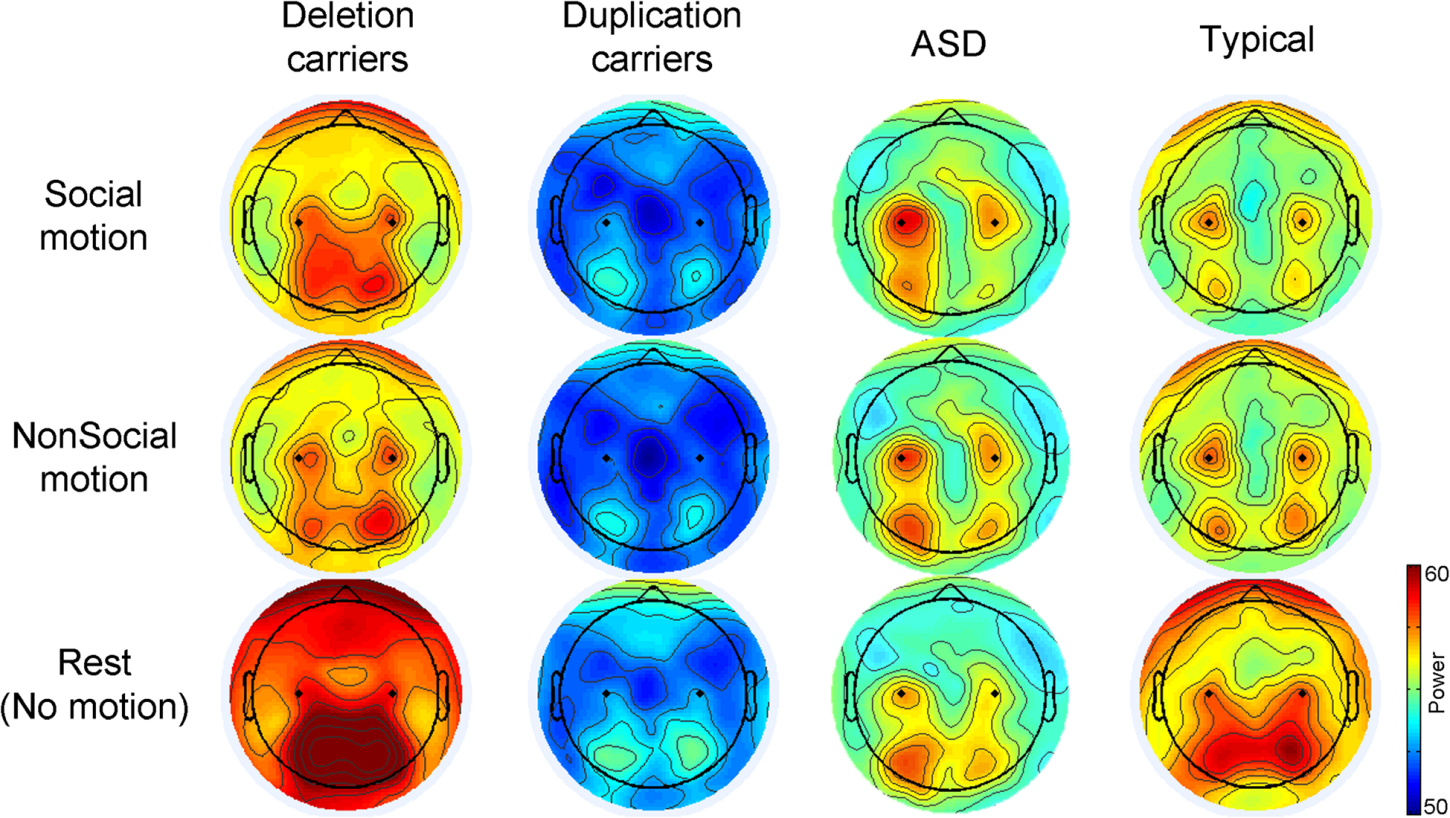
Topographic mu power by group. Power distribution at 10.5 Hz by stimulus condition (social, nonsocial, rest). C3 and C4 electrode locations are indicated by *black dots*

Topographic maps representing group differences in power are presented in Fig. 3 for each stimulus context (collapsed across stimulus environment). In the final model 1, all fixed effects were significant (*p* < 0.05, uncorrected) with the exception of a main effect of the group. Model 1 results are reported in Table 2. Similar to model 0, age significantly contributed to the model, indicating increased mu attenuation for older individuals (0.016 more mu attenuation for every year older than 9 years). Main effects of context and environment indicated more mu attenuation for nonsocial (compared to social) conditions and live action (compared to computer generated) conditions. These effects were modulated by a context × environment interaction, indicating that computer-generated conditions elicited more mu attenuation for nonsocial conditions, *t* (10000) = 4.43, *p* < 0.0001.

**Table 2:** Model 1 results

Fixed effects	*F*	*df*_between_	*df*_within_	*p* value
Context	10.54	1	10,000	0.0012
Environment	10.75	1	10,000	0.0010
Context × environment	9.3	1	10,000	0.0023
Group	0.98	3	42	0.4129
Group × context	9.45	3	10,000	<.0001
Group × environment	5.45	3	10,000	0.0010
Group × context × environment	3.46	3	10,000	0.0156
Age	7.97	1	31.9	0.0081

**Fig. 4:**
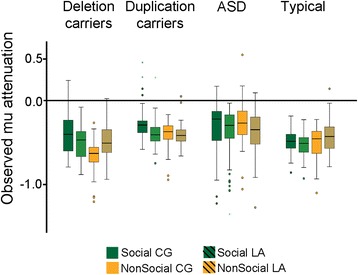
Observed mu attenuation by group. Mu attenuation is depicted as the mean log of the ratio of power in the mu frequency (8–13 Hz) during social (*green*) and nonsocial (*orange*) conditions, separately for live action (*LA*, *solid*) and computer-generated (CG, *striped*) stimuli environments. *Box plots* represent the distribution of mu attenuation across trials as the lower and upper quartiles (*black line* = median). *Whiskers* represent 1.5 times the box height, and *dots* and *asterisks* represent outliers

The primary aim of this paper was to address group differences across conditions. Figure 4 depicts observed mu attenuation by group across all conditions (context × environment). There was no main effect of a group, indicating that each of the four groups had overall similar levels of mu attenuation. However, we observed a significant interaction between group and context with three different patterns of relative mu attenuation between social and nonsocial motion. First, only the typical group elicited the predicted pattern with more mu attenuation to social than nonsocial conditions, *t* (10,000) = 2.30, *p* = 0.022. Second, both of the *16p11.2* carrier groups exhibited the opposite pattern of mu attenuation, such that these groups elicited more mu attenuation to nonsocial than social conditions [deletion carriers, *t* (10,000) = 4.92, *p* < 0.0001; duplication carriers *t* (10,000) = 2.00, *p* = 0.046]. Third, the ASD group did not differentiate between conditions, *t* (10,000) = 1.43, *p* = 0.15.

The group × environment interaction was qualified by the three-way interaction between group, context, and environment. This interaction indicated that the effect of more social mu attenuation than nonsocial for the typical group was primarily driven by differences within the live action environment, *t*(10,000) *=* 2.80, *p* = .0051, but not the computer-generated environment, *t* (10,000) = 0.45, *p* = 0.65. The opposite was true for the deletion carriers, in which the nonsocial context differences (more nonsocial mu attenuation than social) were primarily driven by differences within the computer-generated environment, *t* (10,000) = 6.52, *p* < 0.0001, but not the live action environment, *t* (10,000) = 0.38, *p* = 0.70. Duplication carriers and the ASD group did not exhibit context discrimination differences between environments.

### Single-trial analysis (model 2)

Results for model 2 are reported in Table 3, and parameter estimates are reported in Table 4. Negative parameter estimates reflect more mu attenuation. Parameter estimates are described in relation to the intercept (i.e., all fixed effects at 0), which represents the expected mu attenuation for a 9-year-old in the typical group during the 30th epoch in the live action nonsocial condition. For example, model 2 estimated 0.0569 more mu attenuation in the social context and 0.0072 less mu attenuation in the computer-generated environment for a 9-year-old typical subject. Similar to model 0 and model 1, age was associated with increased mu attenuation for individuals older than 9 years of age, consistent with prior research [1, 2, 24–30, 37].

**Table 3:** Model 2 results

Fixed effects	*F*	*df*_between_	*df*_within_	*p* value
Context	6.74	1	10,000	0.0094
Environment	9.38	1	10,000	0.0022
Context × environment	4.41	1	10,000	0.0357
Group	0.97	3	42.1	0.418
Group × context	8.41	3	10,000	<.0001
Group × environment	4.78	3	10,000	0.0025
Group × context × environment	3.24	3	10,000	0.0213
Age	8.6	1	32.7	0.0061
Time	101.57	1	10,000	<.0001
Time × context	0.02	1	10,000	0.8926
Time × environment	5.58	1	10,000	0.0182
Time × context × environment	0.47	1	10,000	0.4908
Time × group	9.97	3	10,000	<.0001
Time × group × context	2.35	3	10,000	0.0705
Time × group × environment	1.84	3	10,000	0.1377
Time × group × context × environment	1.48	3	10,000	0.2181

**Table 4:** Model 2 estimates

Parameter Estimates	Estimated additional categorical effect	Comparison categorical effect	Estimate	Standard error
Intercept			−0.1886	0.2293
Context	Biological	Nonbiological	−0.0569	0.0218
Environment	Computer generated	Live action	0.0072	0.0222
Context × environment	Biological, computer generated	Biological, live action	0.0351	0.0310
Group	Deletion	Typical	0.0087	0.1603
	Duplication	Typical	0.1623	0.1655
	ASD	Typical	0.1223	0.1806
Group × context	Biological, deletion	Biological, typical	0.0637	0.0335
	Biological, duplication	Biological, typical	0.0935	0.0335
	Biological, ASD	Biological, typical	0.0897	0.0400
Group × environment	Computer generated, deletion	Live action, typical	−0.1006	0.0348
	Computer generated, duplication	Live action, typical	0.0634	0.0345
	Computer generated, ASD	Live action, typical	0.0454	0.0425
Group × context × environment	Biological, computer generated, deletion	Biological, live action, typical	0.1045	0.0480
	Biological, computer generated, duplication	Biological, live action, typical	−0.0328	0.0476
	Biological, computer generated, ASD	Biological, live action, typical	−0.0501	0.0576
Age			−0.0164	0.0056
Time			0.0048	0.0009
Time × context	Biological	Nonbiological	−0.0001	0.0013
Time × environment	Computer generated	Live action	−0.0015	0.0014
Time × context × environment	Biological, computer generated	Biological, Live action	−0.0014	0.0018
Time × group	Deletion	Typical	0.0017	0.0014
	Duplication	Typical	−0.0020	0.0014
	ASD	Typical	−0.0051	0.0017
Time × group × context	Biological, deletion	Biological, typical	−0.0019	0.0020
	Biological, duplication	Biological, typical	0.0034	0.0020
	Biological, ASD	Biological, typical	−0.0001	0.0025
Time × group × environment	Computer generated, deletion	Live action, typical	−0.0010	0.0021
	Computer generated, duplication	Live action, typical	−0.0007	0.0021
	Computer generated, ASD	Live action, typical	0.0039	0.0026
Time × group × context × environment	Biological, computer generated, deletion	Biological, live action, typical	0.0046	0.0028
	Biological, computer generated, duplication	Biological, live action, typical	−0.0006	0.0028
	Biological, computer generated, ASD	Biological, live action, typical	−0.0018	0.0035

Main effects of condition and group estimated at epoch 30 were similar to the overall effects of model 1 with one exception. Unlike model 1, which predicted that more mu attenuation for the social live action condition, model 2 predicted no difference between live action and computer-generated environments, *t* (10,000) = 0.36, *p* = 0.72. This suggests that model 1 indicated that differences in mu attenuation for certain environments in the typical and deletion carrier groups may resolve over the course of the experiment by epoch 30, such that there is no longer a difference in mu attenuation by environment.

**Fig. 5:**
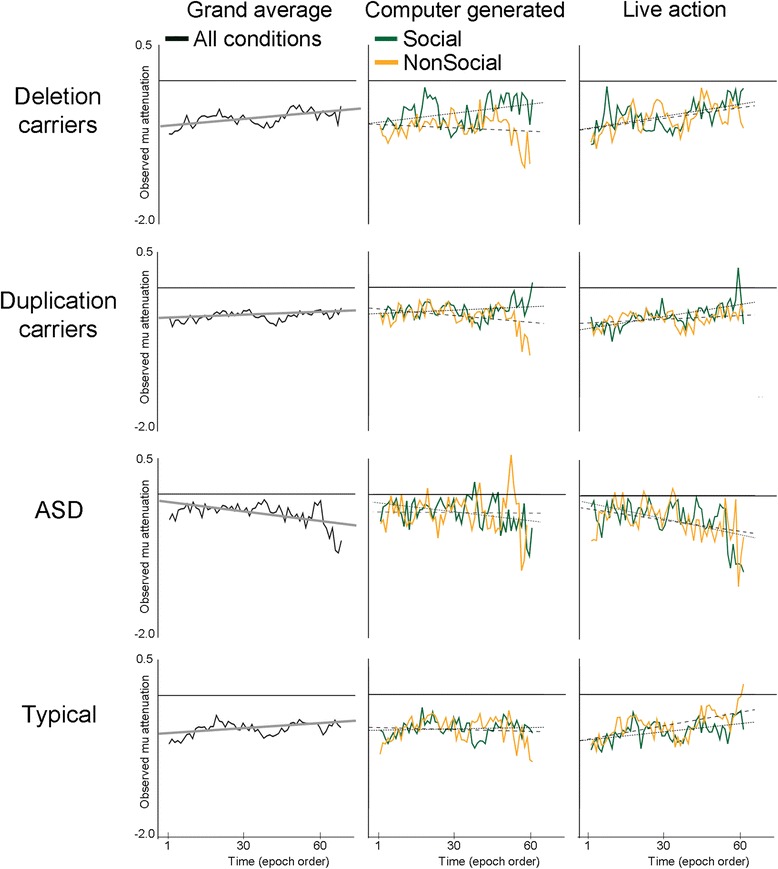
Modulation of mu attenuation over time for *16p11.2* carriers and typical individuals. Observed mu attenuation is plotted for deletion, duplication, ASD, and typical groups across the course of video exposure. Grand average mu attenuation is plotted in the first column in *black* (regression line in *gray*). Social (*green*) and nonsocial (*orange*) mu attenuation is plotted in the second and third columns for computer-generated and live action environments, respectively. Regression lines are plotted for social (*small dashed*) and nonsocial (*large dashed*)

A main effect of time indicated that for subsequent epochs, the mu attenuation intercept (i.e., typical 9-year-old watching a live action nonsocial video) was expected to decrease or lessen. However, as described by the significant group × time interaction, the rate of mu attenuation lessening differed by group. The first column in Fig. 5 depicts this effect as the grand average of observed mu attenuation over epochs for each of the groups. Compared to the typical slope (0.0048), mu attenuation decreased over time more rapidly for the deletion carriers (slope = 0.0028) and less rapidly for the duplication carriers (slope = .0065). In contrast to the other three groups, the ASD slope increased in mu attenuation (slope = −0.00022), suggesting that the neural network supporting motion perception may be increasing efficiency over the course of the videos.

Although the time × group × context interaction was marginally significant (*p* = .071, see Table 3), the second and third columns in Fig. 5 depict the change in mu attenuation for social and nonsocial conditions for computer-generated and live action environments, respectively. These plots highlight that group differences in context discrimination are more apparent during increased exposure to the stimuli (e.g., after epoch 30 of video viewing). Importantly, this figure also illustrates that context discrimination can largely be described as the relative rate of mu attenuation lessening for social and nonsocial motion. For instance, model 1 indicated larger context discrimination for the deletion carriers in the computer-generated motion. Looking at the patterns of mu attenuation over time, this effect is largely driven by the rapid decreasing mu attenuation of the social condition, whereas mu attenuation for the nonsocial condition does not lessen much over time.

**Fig. 6:**
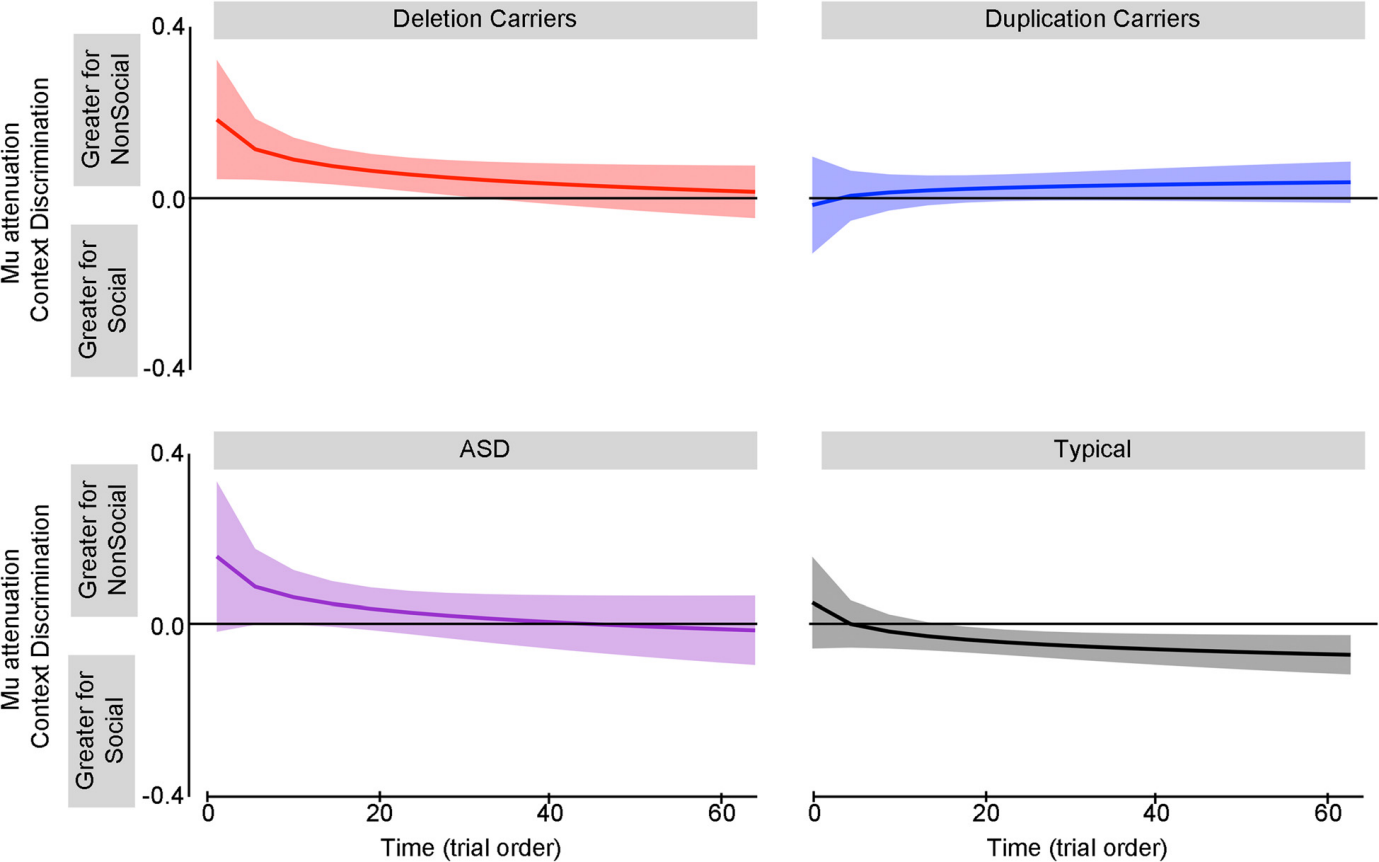
Relative mu attenuation between social and nonsocial conditions by group. Observed mu attenuation differences between social and nonsocial conditions are plotted for deletion carriers, duplication carriers, ASD, and the typical group across the course of video exposure. The *line* represents the group mean and the *shaded values* represent the 95 % confidence interval. Positive values indicate more mu attenuation to the nonsocial context. Negative values indicate more mu attenuation to the social context

To fulfill our objective of characterizing dynamic changes in mu attenuation related to social perception, we conducted a series of pairwise comparisons between social and nonsocial context for each group every 5 epochs from epoch 1 to epoch 60. Figure 6 plots mu attenuation modulation as a difference score, such that negative values represent more mu attenuation to social than nonsocial context. Each group exhibited a unique pattern of mu attenuation related to context discrimination. Both deletion and duplication carriers exhibited more nonsocial than social mu attenuation. This pattern was consistent for deletion carriers from trial 1 [*t*s (10,000) = 2.01–4.22, *p*s < 0.05] until epoch 60, *t* (10,000) = 1.73, *p* = 0.083. However, the duplication carriers did not begin discriminating contexts until epoch 30 and continued to exhibit more mu attenuation for nonsocial conditions until the end of the video, *t*s (10,000) = 2.09–2.92, *p*s < 0.05. In contrast to the carrier groups, the typical group exhibited greater mu attenuation for social conditions at epoch 25, *t*s (10,000) = 2.0–2.6, *p* < 0.05. Unlike all other groups, the ASD group did not exhibit mu attenuation context discrimination at any epoch, indicating equivalent levels of mu attenuation for social and nonsocial conditions.

### Post hoc comparisons of *16p11.2* carriers

To better understand how these patterns may be related to heterogeneity within these unique samples, we conducted a series of post hoc ANOVAs to determine if patterns of mu attenuation are consistent for *16p11.2* carriers between (1) children and adults and (2) between *16p11.2* carriers with and without an ASD diagnosis. While these comparisons may be subjected to type I and II errors due to small sample size, these analyses were meant to qualitatively support model 1 and model 2 predictions. The patterns of mu attenuation for these comparisons are depicted in Additional file 1: Figure S1, Additional file 2: Figure S2, and Additional file 3: Figure S3.

First, child deletion carriers [*n* = 9, *F* (1, 1861) = 16.28, *p* < .001] and adult duplication carriers [*n* = 5, *F* (1, 1111) = 15.19, *p* < .001] exhibited the overall group pattern exhibited with greater mu attenuation for nonsocial (compared to social) conditions. There was no difference between context for adult deletion carriers [*n* = 7, *F* (1, 740) = 1.87, *p =* .17] or child duplication carriers [*n* = 3, *F* (1, 1567) = .002, *p* = .96]. However, visual inspection of the dynamic mu attenuation patterns across time (Additional file 2: Figure S2) suggests that the adult deletion carriers exhibited greater mu attenuation for the nonsocial context. Unlike the other *16p11.2* carrier age groups, child duplication carriers exhibited a pattern of mu attenuation changes that is more similar to the ASD group (no context discrimination but trending towards social greater than nonsocial).

Second, neither deletion carriers (*n* = 3) nor duplication carriers (*n* = 3) with an ASD diagnosis exhibited context discrimination, *F*s < .31, *p*s > .58, similar to the pattern of mu attenuation for the ASD (idiopathic, non-*16p11.2* carrier) group. Visual inspection of the dynamic mu attenuation patterns across time (Additional file 3: Figure S3) indicates a large amount of variability for the deletion carriers with ASD, consistent with prior work looking at social motion perception in ASD [3, 5–14, 22, 31]. These results suggest that social motion mu attenuation within *16p11.2* carriers is associated with ASD and the concomitant social cognitive phenotype.

## Discussion

This study sought to characterize how the functional social brain phenotype of a preliminary sample of individuals with *16p11.2* CNVs compares to typical and ASD individuals. As predicted, typical individuals exhibited greater mu attenuation for social motion relative to nonsocial motion, particularly within the live action environment. Contrary to this typical pattern of mu attenuation, we found that both *16p11.2* CNV duplication and deletion carriers exhibited greater mu attenuation for nonsocial motion relative to social motion. This pattern differed from individuals with ASD, who elicited equivalent levels of mu attenuation across conditions, consistent with prior work indicating reduced mu attenuation in ASD in response to social motion compared to controls [15, 32–35, 37, 38].

An innovation and benefit of our analytic strategy was the use of single-trial analysis to assess ongoing dynamic changes over the course of the motion observation, designating two important discoveries. First, the results confirm that mu attenuation is not static but rather decreases or lessens across exposure to motion stimuli for the typical and *16p11.2* CNV groups. Specifically, this decrease in mu attenuation occurs more rapidly for the deletion carriers than either the duplication carriers or the typical group, whereas mu attenuation increases over time for the ASD group. The degree to which the mu rhythm attenuates overexposure to stimuli may implicate different patterns of general habituation or information processing. Recent evidence suggests that *16p11.2* CNVs exhibit a dose-dependent effect on both cortical and subcortical structures [46], such that compared to controls, brain volume is increased for deletion carriers and reduced for duplication carriers. Thus, the discordant rates of habituation observed among deletion and duplication carriers may correspond to differences in brain structure and/or circuitry.

Second, the single-trial analysis indicated that the relative patterns of mu attenuation between social and nonsocial motion vary differently for each group over time. Specifically, these patterns indicated three unique atypical patterns of mu attenuation: overall increase for nonsocial (*16p11.2* deletion), mid-experiment increase for nonsocial (*16p11.2* duplication), and no motion discrimination (ASD). The duplication carriers and the typical group exhibited proportionally equivalent mu attenuation during the initial portion of social and nonsocial videos. Subsequently, after 25–30 epochs, the modulation of mu attenuation diverged for these two groups, such that the duplication carriers followed an atypical trajectory with greater mu attenuation for nonsocial conditions. It may be that while social stimuli are initially similarly salient for duplication carriers and typical individuals, the maintenance of that salience at the neurophysiological level differs, resulting in a rapid decrease in neurophysiological processes devoted to social information processing.

In contrast, discrimination is stable across exposure for the *16p211.2* deletion carriers, albeit with an atypical pattern of mu attenuation (i.e., greater attenuation for nonsocial). While both CNV groups exhibit greater mu attenuation for nonsocial relative to social motion, this overall pattern of discrimination occurs during the later exposure trials for the duplication carriers. This finding reflects different temporal processing trajectories, which may be driven by unique underlying neural systems for integrating social information. One possible explanation may be that the CNV groups differ in level of motivation to engage with social stimuli. Considering the rapid rate of mu attenuation reduction during the observation of social motion for the deletion group (see Fig. 4), it is possible that deletion carriers may fail to truly engage with the social stimuli, driven perhaps by less motivation or salience.

However, within this preliminary study, individuals with *16p11.2* CNVs and a diagnosis of ASD have divergent trajectories (compared to *16p11.2* carriers without ASD), more aligned with the individuals with idiopathic ASD. In other words, the functional social brain phenotype associated with ASD supersedes the unique *16p11.2* deletion and *16p11.2* duplication phenotype. Each unique atypical pattern may correspond to different aspects of social cognitive impairments. For instance, individuals with ASD do not exhibit social and nonsocial motion discrimination, supporting theories of complex information dysfunction [42, 52]. In comparison, the CNV carriers without ASD respond more strongly to nonsocial motion, possibly indicative of a reduction in salience or motivation for social information or increased salience of nonsocial information. Considering the social motion perception pattern for individuals with *16p11.2* CNVs and ASD, it is likely that concomitant features of social cognition (e.g., emotion recognition, social motivation) more closely resemble ASD impairments [53–56].

Given the relationship between *16p11.2* CNV and neurodevelopmental disorders, a developmental model is critical. Prior work indicates a diagnosis-independent emergence of mu attenuation across development [37], but both aspects of time (developmental stage and temporal processing) are critical in understanding how the functional social brain phenotype emerges. While both duplication and deletions carriers showed specific effects of neural responses to social motion, it is possible that the observed variability within our carrier groups may have been related to “second hits” in the genome or to genetic variation at other loci [57, 58]. In the same vein, the “idiopathic” ASD group likely reflects a combination of etiologies, which may separately confer varying effects on the neurophysiological presentation rendering increased variability within that group.

The three primary strengths of this study include the use of careful molecular subtyping as a “genetics-first” approach [59], neurodevelopmental comparisons between CNV and ASD groups, and the unique statistical methods that permitted the dynamic measurement of social perception. Nevertheless, this study is limited by sample size and age range of the individuals. Our post hoc comparisons suggest that social perception *16p11.2* CNV patterns are fairly consistent across development, despite the large age range. Considering the rarity of this group of individuals, the results are informative as a preliminary investigation of the *16p11.2* CNV social brain phenotype. As genetic testing becomes more commonplace, the percentage of known individuals with *16p11.2* CNVs will increase. While investigating this CNV and other rare variants is a long-term process, these results justify continuing to use neuroimaging in order to elucidate the underlying pathogenic mechanisms involved with unique molecular subtypes.

## Conclusions

In conclusion, our findings strongly support the notion that *16p11.2* carriers exhibit atypical social perception, similar to, yet distinct from, ASD. Future research should continue to address clinical and behavioral heterogeneity by reducing variance through large, carefully matched populations. The process by which these patterns emerge in relation to developmental age may be of particular interest, as well as how mu attenuation is impacted by clinical interventions promoting social development. Additional considerations for individual differences would benefit from the inclusion of robust behavioral measures of social cognition previously linked to spectral power in the mu rhythm, such as imitative ability [60].

## Additional files

Additional file 1: Figure S1. Post hoc comparisons of age and ASD Diagnosis within *16p11.2* carriers. Error bars represent 95 % confidence interval of the mean. Significant comparisons are noted with an asterisk. Additional file 2: Figure S2. Relative mu attenuation between social and nonsocial conditions over time for *16p11.2* carriers by age group. Observed mu attenuation differences between social and nonsocial conditions are plotted for deletion carriers and duplication carriers, separately for children (younger than 18 years, dash-dot line) and adults (18 years and older, solid line). The line represents the group mean and the shaded values represent the 95 % confidence interval. Positive values indicate more mu attenuation to the nonsocial context. Negative values indicate more mu attenuation to the social context. Black arrows indicate the point in time by which model 2 indicated significant context differences. Gray arrows with an “X” indicate the point in which there are no longer a significant context difference. Additional file 3: Figure S3. Relative mu attenuation between social and nonsocial conditions over time for *16p11.2* carriers by ASD diagnosis. Observed mu attenuation differences between social and nonsocial conditions are plotted for deletion carriers and duplication carriers, separately for individuals without an ASD diagnosis (top, solid line) and with an ASD diagnosis (bottom, dashed line). The line represents the group mean and the shaded values represent the 95 % confidence interval. Positive values indicate more mu attenuation to the nonsocial context. Negative values indicate more mu attenuation to the social context. Black arrows indicate the point in time by which model 2 indicated significant context differences. Gray arrows with an “X” indicate the point in which there are no longer a significant context difference.
