# Supplementary figures and images for: Modulation of mu attenuation to social stimuli in children and adults with 16p11.2 deletions and duplications
Source: J Neurodev Disord. 2015 Jul 24;7(1):25. doi: 10.1186/s11689-015-9118-5 (PMC4514956; doi:10.1186/s11689-015-9118-5)

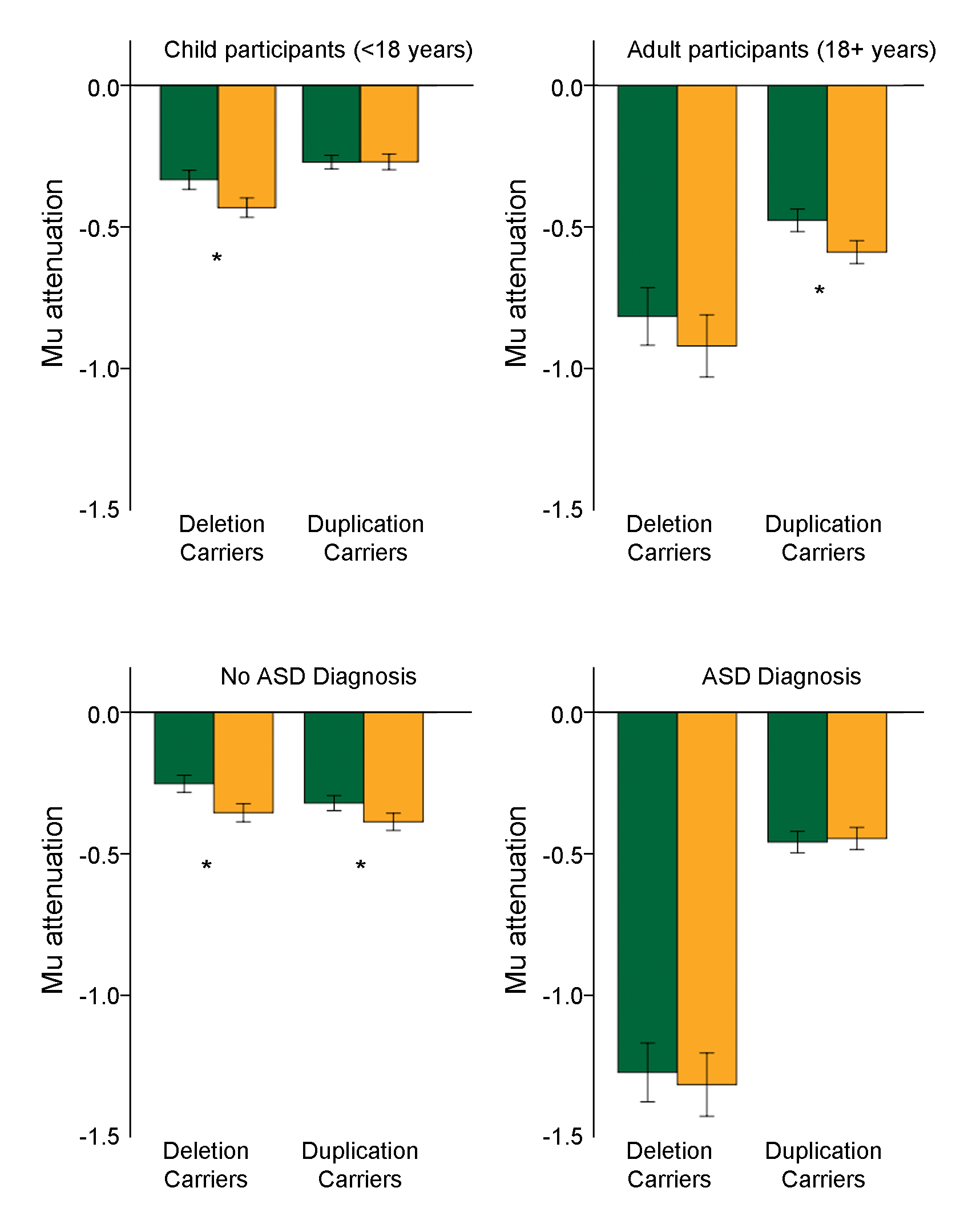

Supplement: Additional file 1: Figure S1. — Post hoc comparisons of age and ASD Diagnosis within 16p11.2 carriers. Error bars represent 95 % confidence interval of the mean. Significant comparisons are noted with an asterisk. [file 11689_2015_9118_MOESM1_ESM.tiff]

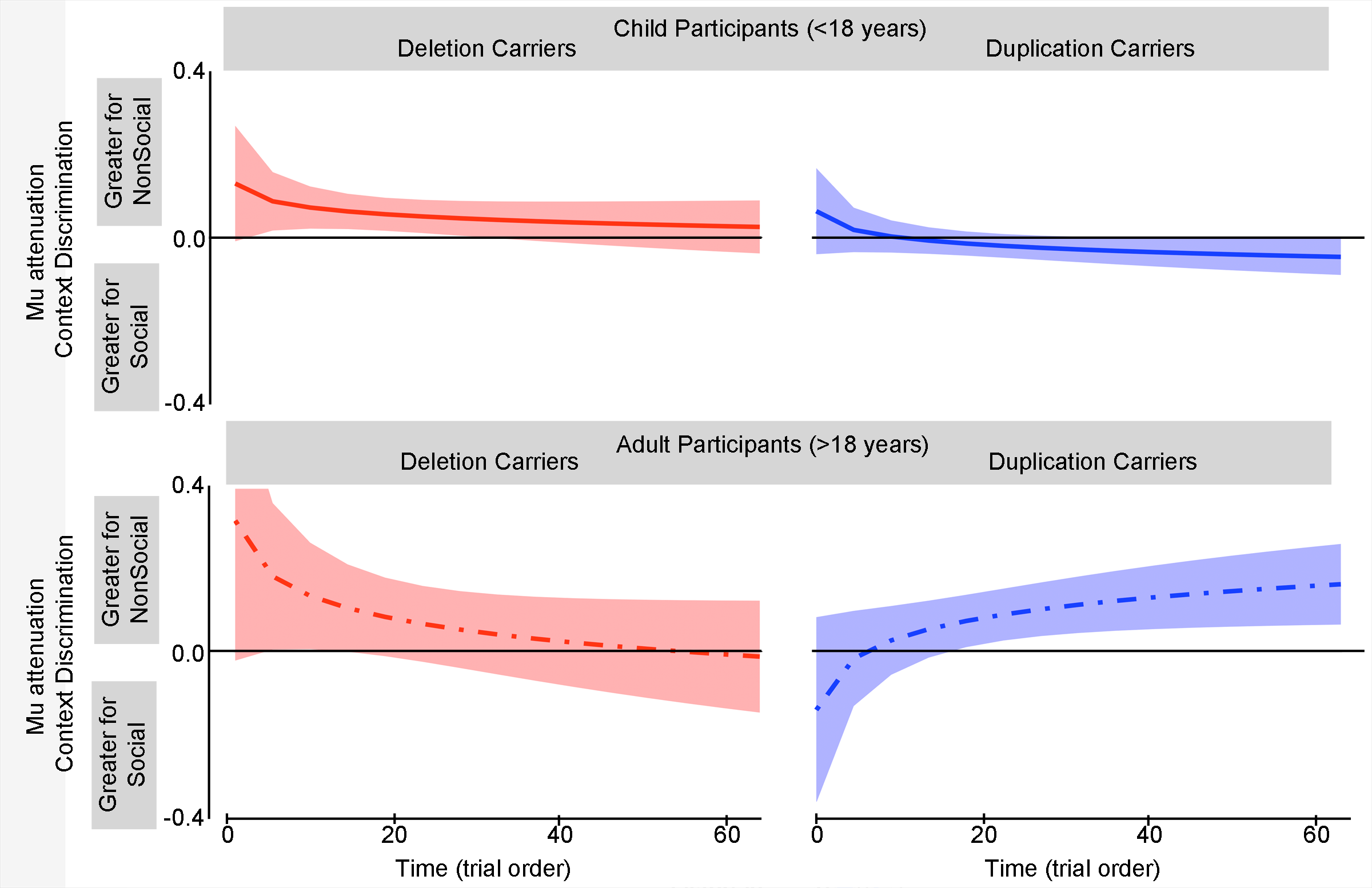

Supplement: Additional file 2: Figure S2. — Relative mu attenuation between social and nonsocial conditions over time for 16p11.2 carriers by age group. Observed mu attenuation differences between social and nonsocial conditions are plotted for deletion carriers and duplication carriers, separately for children (younger than 18 years, dash-dot line) and adults (18 years and older, solid line). The line represents the group mean and the shaded values represent the 95 % confidence interval. Positive values indicate more mu attenuation to the nonsocial context. Negative values indicate more mu attenuation to the social context. Black arrows indicate the point in time by which model 2 indicated significant context differences. Gray arrows with an “X” indicate the point in which there are no longer a significant context difference. [file 11689_2015_9118_MOESM2_ESM.tiff]

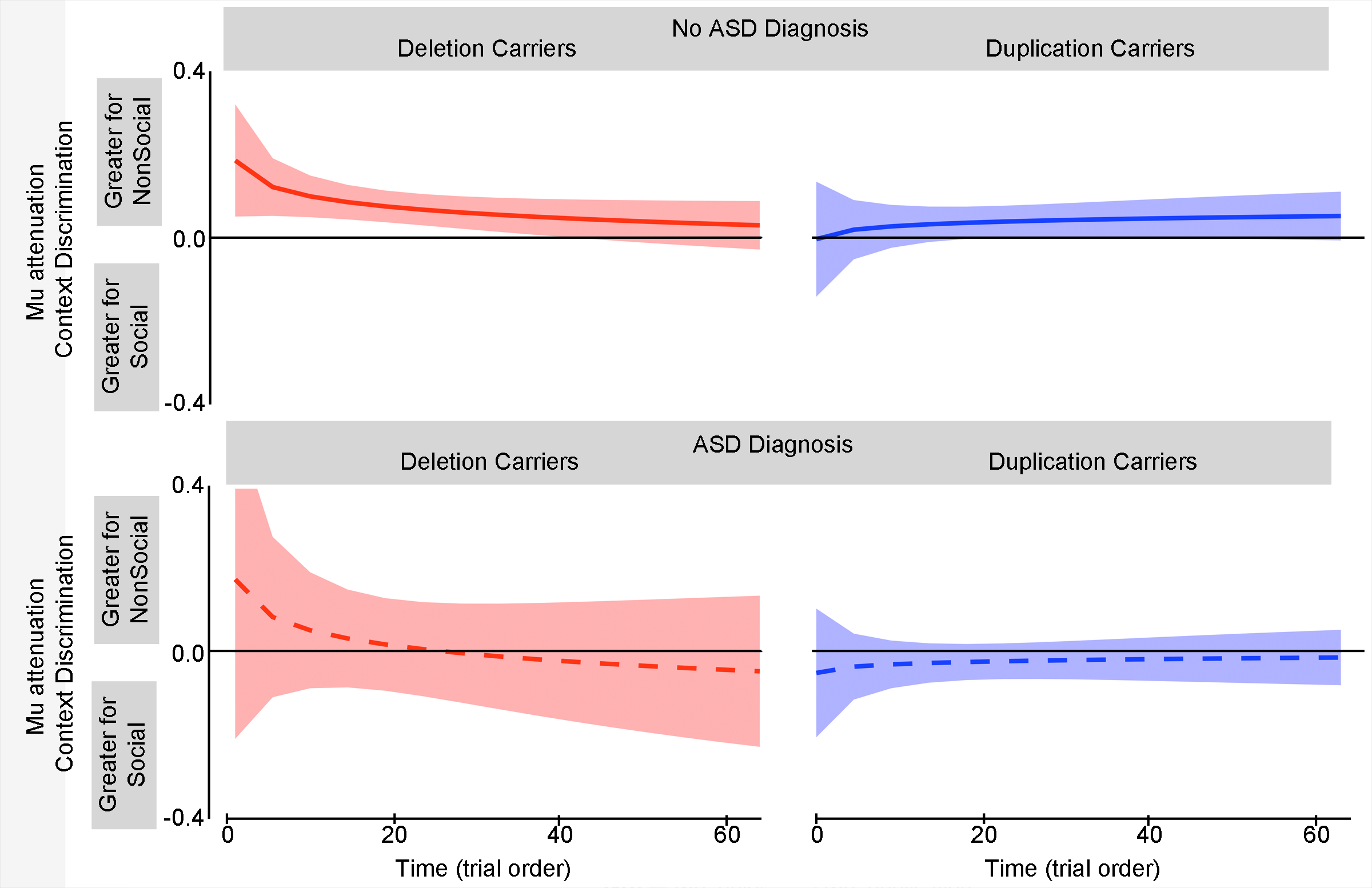

Supplement: Additional file 3: Figure S3. — Relative mu attenuation between social and nonsocial conditions over time for 16p11.2 carriers by ASD diagnosis. Observed mu attenuation differences between social and nonsocial conditions are plotted for deletion carriers and duplication carriers, separately for individuals without an ASD diagnosis (top, solid line) and with an ASD diagnosis (bottom, dashed line). The line represents the group mean and the shaded values represent the 95 % confidence interval. Positive values indicate more mu attenuation to the nonsocial context. Negative values indicate more mu attenuation to the social context. Black arrows indicate the point in time by which model 2 indicated significant context differences. Gray arrows with an “X” indicate the point in which there are no longer a significant context difference. [file 11689_2015_9118_MOESM3_ESM.tiff]
